# The experience of long stay in high and medium secure psychiatric hospitals in England: qualitative study of the patient perspective

**DOI:** 10.1186/s13033-020-00358-7

**Published:** 2020-03-30

**Authors:** Jessica Holley, Tim Weaver, Birgit Völlm

**Affiliations:** 1 Research Fellow, Middlesex University, London, UK; 2 Associate Professor of Mental Health Research, Middlesex University, London, UK; 3 Professor in Forensic Psychiatry, University of Nottingham and Nottinghamshire Healthcare NHS Foundation Trust, Nottingham, UK

**Keywords:** Forensic mental health, Length of stay, Long stay patients, Mentally disordered offenders, Secure care, Qualitative interviews, Thematic content analysis, Narrative analysis

## Abstract

**Background:**

Some forensic patients in England remain in secure care for long, possibly unnecessarily prolonged, periods, raising significant ethical and resource issues. Research focused on the patients in secure care has examined quality of life and service provision but not the perspectives of patients experiencing long stays. This study explored how long stay patients experience secure care, what factors they felt influenced long stay, and its impact upon treatment engagement and motivation to progress.

**Methods:**

Embedded within a larger epidemiological study, we conducted semi-structured interviews with a purposive sample of forty long stay patients from two high and six medium secure hospitals. Long stay was defined as a 5 years stay in medium secure care *or* 10 years in high secure care, *or* 15 years in a combination of high and medium secure. Transcripts were subject to thematic analysis, and narrative analysis at individual case level to explore the relationship between emergent themes.

**Results:**

Four themes emerged illustrating participants’ attribution, outlook, approach, and readiness for change. A typology of four long stay stances was developed (dynamic acceptance, dynamic resistance, static acceptance, static resistance). These illustrate differences in the extent to which participants believed being in secure care helped them to get better, and actively work towards progression and leaving secure care. There were considerable differences in how patients adopting these stances attributed the reasons for their long stay, they viewed their future, and their motivation to progress. Negative perceptions arose from excessive restrictions, treatment repetition and changes in therapeutic relationships, leading some patients to exhibiting tokenistic engagement and low motivation to progress.

**Conclusions:**

Planning care for long stay patients in secure psychiatric settings should take account of the differing stances patient’s adopt towards engagement and progression. Service providers should be mindful of these stances and provide patients with individualised opportunities to progress through the secure care treatment pathway, avoiding treatment repetition and maintaining continuity in key professional relationships. Refocusing on quality of life may be appropriate for some long-term patients who are unwilling or unable to move on. For some long-term patients, purpose designed long stay setting may be appropriate.

## Background

Forensic psychiatry is concerned with the management of patients with psychiatric disorders, most of whom have committed an offence serious enough to require detention in a secure setting. In the UK, patients are admitted to secure forensic services because they have a history of serious violence and pose a serious or grave risk to the public [7]. The aims of such detention are to (a) provide care and treatment designed to improve mental health and facilitate recovery, and (b) provide protection to the public by reducing the potential risk posed by the patient [20].

In England, inpatient forensic psychiatric care is available at three high security National Health Service (NHS) hospitals and, at the time of this study, 57 medium secure units, some run by NHS Trusts and others by independent providers. More recently a smaller number of low secure facilities have been added. Patients may be moved between hospitals of different levels of security.

Concern has been expressed over the length of time some patients remain in a secure care setting. In England, length of stay in forensic psychiatric settings greatly exceeds that in general psychiatric services [33] and often also exceeds length of imprisonment for the same offence [39]. As many as 27% of patients in both high and medium secure settings stay at least 10 years [31] and a substantial proportion of forensic patients in medium secure settings stay longer than the 2 years originally recommended for such units [11, 26]. Indeed, a large epidemiological study to which the work reported in this paper was linked [40] found that 22% of patients in high security and 18% in medium security were “long stay” (which we defined as having stayed 5 years in medium secure care or 10 years in high secure care, or 15 years in a combination of high and medium secure settings). There is also concern that these long stays are frequently cared for at too high levels of security [2, 38, 29].

Secure forensic services are expensive, and unnecessarily long stays in secure care raise significant resource issues [10]. Furthermore, the tension between the requirements of treatment and security has long been recognised. These services are extremely restrictive for those detained within them [20] and it is well established that the highly restrictive nature of secure care services can impact negatively upon quality of life [34].

It is now widely accepted that obtaining the views of the recipients of health care is an essential element in the evaluation of mental health services [9]. However, whereas information is available on the needs of forensic service users from a staff perspective (e.g. [19]), on measured quality of life (e.g. [41] and on recovery [14]), only a limited number of qualitative studies have explored the experience of secure care from the patients’ perspective. Some of these studies have examined service satisfaction (e.g. [13, 8]), patients’ experiences of daily routines and their use of time (e.g. [12, 36]), and the attitudes and experiences of older patients in secure care [44], but none have specifically focused on how those with prolonged stays in a secure setting experience their daily life.

From a clinician’s point of view, discharge from forensic care depends on a number of factors including the assessment of the patient’s risk upon and their progress with treatment and rehabilitation. Reasons for delayed discharge are likely to include poor response to treatment, ongoing safety issues, and lack of a suitable step-down facility. A recent review found the factors most frequently associated with longer stay included seriousness of the index offence, history of psychiatric treatment, cognitive deficit, severity of illness, diagnosis of a psychotic disorder and history of violence [18]. Whereas qualitative studies have shown, for example, that forensic patients can appreciate the therapeutic benefit of having a ‘safe space’ in order to progress [24], there is little information on how long stay patients view the reasons for their long stay in secure settings or how they feel about possible progression through the system, perhaps to a lower level of security or even to discharge to the community. Arguably, awareness of these perceptions, ambitions and motivations would allow services to be better tailored to the needs of this specific client group.

We sought to address this gap in the research evidence by investigating how long stay patients perceive (a) factors that they felt had influenced their length of stay in secure care, particularly where detention extends well beyond the completion of treatment, (b) their day to day experience of their long stay, and (c) the impact that longer lengths of stay have upon their engagement in treatment and motivation to progress.

## Methods

### Participants and settings

We interviewed long stay patients purposive sampled from 2 high and 6 medium secure UK hospitals. For the purpose of this study, long stay was defined as > 10 years in a high secure setting, > 5 years in a medium secure setting, or > 15 years in a combination of both. Our initial target was to interview 30 long stay patients, but we increased our final sample size to achieve data saturation and represent key sampling criteria. A purposive sampling strategy was adopted [27] to achieve an interview sample that exhibited the necessary range and diversity in terms of the aims of the study. This involved the application of formal sampling criteria for both site and participant selection.

To sample sites, our starting point was the 26 units participating in the linked epidemiological study of long stay patients [40]. These represented two of the three high secure hospitals and a stratified sample of 23 of all 57 medium secure units in England at time of the study. The two high security units were automatically sampled. The medium secure units were classified by provider type (NHS or independent) with six units (3 NHS, 3 independent) purposively selected from those with sufficient numbers of long stay patients to recruit a sample (i.e. having > 10 such patients). Final selection of sites ensured representation of different geographical regions, and those holding populations of female long stay patients.

To sample participants, an anonymised list of patients at the sample sites was first generated. Potential participants were stratified by gender and length of stay (above and below median length of stay). Where possible we included at least one female patient at each site, with the final sample purposively selected to include range and diversity in terms of the following secondary sampling criteria: Mental Health Act (MHA) section (37 (hospital order); 37/41 (hospital order with restrictions); 47/49 (transfer from prison to hospital); 3 (treatment order)); age (< 50, > 50 years); ethnicity (White British/non-White-British); clinical diagnosis (schizophrenia, personality disorder, learning disability); index offence; offence history (one-off/repeated offending) and admission source (prison, high and medium secure settings).

At each site we initially sampled up to 10 long stay patients in anticipation of some attrition and refusal to participate. Initially, each patient’s lead clinician responsible for his or her care was asked whether there were clinical issues that might prevent participation. If not, the patient’s named nurse gave the patient an information sheet and asked whether they would be willing to participate in principle. If the patient agreed, written informed consent was requested. Refusal at either point resulted in no further contact from the research team. Where available substitutes were approached in order to achieve our sample size and characteristics.

### Design and procedure

This qualitative study adopted a constructivist epistemological perspective. The constructivist position holds that the way people understand and perceive the world is constructed through their personal experiences, relationships and social interactions [3]. The study focussed on a sensitive exploration of the experience and perspective of individuals who stay in secure settings for long periods of time. Each consenting patient took part in a semi-structured interview in which participants were asked a series of questions that explored reasons for their long stay, their current situation and their feelings about their future progression through the system. All interviews were conducted by JH and were digitally recorded. An interview guide was created following a review of the literature and discussion within a service user reference group, and then refined during the pilot interviews. Probes and follow up questions were used to encourage self-reflection by the interviewee. The interview agenda and topic guide are available as Additional file 1). The interviewer was free to vary the order and wording of questions for the purpose of rapport and clarity of meaning. Interviews typically lasted around 50 min (range 30 min to 1¼ h).

### Analysis and interpretation

Interview recordings were transcribed verbatim and uploaded into NVivo qualitative data analysis software (QSR International Pty Ltd. Version 11, 2015). Analysis then took place in two phases. In the first phase, a thematic content analysis was conducted using a framework approach to organise data into the topic guide’s main areas of enquiry [30]. Within these areas, data were subject to open coding to identify categories that represented key issues discussed by participants. Each transcript was first read several times to get an overall sense of the material. Any places in the text that had relevance to at least one of the study’s aims were marked and moved to a coding sheet. An open code was then assigned that matched the content as closely as possible. These open codes were then organised under higher order headings from which preliminary themes and subthemes were generated within each area of enquiry. After several transcripts were coded, a constant comparative method was used to group and merge common categories together [15] allowing the number of preliminary themes and subthemes to be gradually reduced. Where coded data were revised or new codes identified, previously coded transcripts were re-checked to ensure that these extracts of data had not been missed, and that a consistent approach to coding was employed throughout. The emergent themes were used to inform the second phase of analysis.

In the second phase of analysis, the relationship between the emergent themes at an individual case level was explored using a narrative approach [32]. This was done in order to capture the manner in which events were described and evaluated in telling each participant’s story [1]. This analysis was informed by a constructivist position and allowed distinct patterns to emerge concerning how the participants made sense of the present (their current situation), past (reasons for their long stay), and future (moving on).

All stages of the process were conducted by at least two of the authors; first independently and then by discussion and reaching consensus. Important decisions were taken jointly by all three authors. JH is a research associate, TW is a social scientist in health services research, and BV is a consultant forensic psychiatrist; all authors hold PhD degrees. Our pre-understanding of the subject area derives from our review of the literature, our professional education, and our personal experiences of forensic psychiatry.

## Results

### Description of sample

We conducted a total of 40 interviews. In order to achieve an interview sample representative of characteristics relevant to the research objectives, 125 cases were selected. Of these, 26 had been discharged from the unit and 2 had died. In 6 cases the lead clinician would not permit the patient be interviewed; this was due to the patient’s mental state being deemed too unstable at the time of the research study. Of the remaining 91 approached, 40 gave informed consent and were interviewed while 51 refused.

Of the 40 patients interviewed, the majority were male (n = 34) and of white British ethnicity (n = 30). Their median age was 46 years (range 23–72 years). Eleven patients were recruited from NHS high secure units, seventeen from NHS medium secure units and twelve from independently-run medium secure units. The median overall lengths of stay in secure care for these three sub-groups were 17.3 years, 13.3 years and 16.1 years respectively. Their primary clinical diagnosis included schizophrenia or other psychosis (n = 19), personality disorder (n = 16) and learning disability (n = 5). Their index offences invariably involved major violence or sexual offences, or both; repeated offences were recorded for many (n = 35). Two patients had no recorded offence history. The majority were detained under the UK Mental Health Act on section 37/41 (n = 27), the remainder being detained under section 47/49 (n = 6), section 37 (n = 3) and section 3 (n = 4).

We describe below the findings in relation to the three key foci of the study: patients’ perception of their current situation (the present), reasons for their long stay (the past), and their potential for progression (the future). Four key themes of *attribution*, *outlook*, *approach* and *readiness for change* emerged and are discussed.

### Current situation

Most participants who had moved from a high secure setting to a medium secure unit thought that their current unit had less restrictive regimes, and some felt that they had received better treatment in the latter. There were exceptions, however. Several participants perceived medium secure units to be relatively rule-bound, whereas others who had moved from medium to high secure units felt that daily life was easier because they had less responsibility. The majority thought treatment was better in secure hospitals than in prison, although some felt that prison offered them a greater scope than hospital to organise their own daily routine.

Many participants described what they perceived to be unnecessary restrictions when residing on wards with patients whom they perceived as more unwell and higher risk. Some cited not being able to go on escorted leave as staff time was taken up by needier patients, others described activities being withdrawn following specific incidents or general misuse. These restrictions were a particular issue for older participants on wards that accommodated mostly younger patients.

When considering daily routines and occupation, many participants described the importance of keeping busy. Occupational activities (notably cooking, gym and educational courses) were particularly valued. Others, however, felt that their routines were monotonous and repetitive, with some reporting having undertaken the same activities for many years or that the activities on offer would not necessarily be something that they would find relevant outside secure care. Whereas many described how they found psychological therapies beneficial, there were others who felt the therapies were ineffective and questioned why they were repeating them in the absence of any change in their situation.

With regard to interpersonal relationships, a majority of participants believed that most staff were helpful, caring and had their best interests at heart. Participants with this outlook mostly felt that it was important to actively engage with staff in order to build trust, particularly when they were experiencing problems. One participant positioned himself as a staff member in order to illustrate how his own relationships with staff had helped him to progress:

***“****Basically, support him when he’s doing well, try and play it down when he’s not doing too well… So like, I then flourished.”* (NHS high secure hospital)

Nevertheless, it was common for participants to report having disagreements with staff. This was usually described in terms of differing perceptions of risk, feeling unnecessarily restricted, or a simple clash of personality. Some participants minimised their engagement with staff in order to avoid such disagreements. Staff turnover caused problems for some patients, especially when they experienced issues gaining the trust of staff.

Half of the participants interviewed said they valued their friendships with other patients. Those who did not, however, described getting into arguments or fights and consequently preferred to keep to themselves. Others reported not needing or wanting to make friends, commonly citing perceived differences between themselves and other patients in relation to their index offence, diagnosis, perception of risk, length of stay and source of admission. Some also described how it was difficult to get on with other patients who were a lot younger than they were, citing a lack of common interests. Those who were residing on specific long stay units/wards described getting on with other patients who were of similar age and had similar needs to them. Some participants reported avoiding close friendships because they found that relationships they had built in the past could not be maintained when either they or the other patient moved to another unit.

Two themes arose from participants’ discussions about their current situation. The first was coded as *outlook* (how they view their current situation), which could be positive or negative. Where the outlook was negative, this resonated through all aspects of their day-to-day life in secure care. The second was coded as *approach* (how they manage their current situation), which could be proactive (tending to be self-motivated, engaging with others, and seeking to make the most of their current situation) or passive (tending to lack motivation and not engaging). Some participants had a positive outlook and a proactive approach; for example, they described how keeping busy and taking part in activities and occupations was beneficial to them. In contrast, some had a negative outlook but still adopted a proactive approach; for example, describing the ineffectiveness and pointlessness of activities and/or therapies whilst still believing that taking part would help them progress. In contrast, others who held a negative outlook and took on a passive approach had a tendency not to participate.

### Reasons for long stay

Participants’ accounts varied when explaining why they felt they had stayed so long in secure care, which included why they had or had not moved units. One theme, coded as *attribution*, emerged where a prolonged stay was attributed to personal, interpersonal and/or structural factors, either alone or in combination. It was evident, however, that these accounts were mediated by participants’ differing levels of awareness about their poor mental health or the severity of their offending. A majority of participants attributed their length of stay to events prior to their admission such as the severity of their index offence or their offending history. Those who made these attributions believed their length of stay was justified and within their expectations. While a number of participants attributed progressive moves between wards or units to their engagement with treatment and with staff, others recognised that their length of stay had been extended because of their disruptive behaviour whilst in secure care. These explanations were commonly paired with descriptions of factors that were implicitly or explicitly presented as mitigation, for example in reference to being seriously unwell, ‘on medication’, or on a unit not matched to their needs.

Other participants attributed their length of stay to factors over which they had neither control nor responsibility, notably the structure or organisation of the treatment system. Examples included a change in the clinician responsible for the patient’s care and consequent changes to his or her treatment plan, a change in the system’s requirements for discharge, and the absence of an appropriate facility to move on to. One patient considered the high turnover in clinicians responsible for his care might be due to a shortage of clinicians who were specialised in personality disorder. Some participants acknowledged that their own disruptive behaviour was a factor in their long stay, often resulting in sideways moves between wards or units. However, they often felt that the institution’s response had been disproportionate or reflected a risk-averse approach that restricted their progression, or both.

*“I’ve been ticking all the boxes, you know […] I just have, like, a few minor concerns… they’re trying to exploit it, exaggerate the situation, from these minor things, to make me look bad, you know”* (NHS medium secure site).

### Progression and ‘moving on’

Most participants described ‘moving on’ in terms of a physical progression to a lower security setting or into the community. Some additionally described this in psychological terms such as being able to start again by ‘shaking off’ their offence history. One theme, coded as *readiness for change*, emerged from participants’ discussions about the future and encompassed how the participant felt about moving to a lower level of security. This emerged as a clear dichotomous element in the interview transcripts: participants either felt a very clear readiness for change or they did not.

Several factors were reported to be associated with feeling ready to move on: that their length of stay in secure care had exceeded their original sentence, the sense that their current security level was excessive, and the perception of having made progress through good behaviour or by completing treatments. In this context, it was apparent that a conditional ‘tick-box culture’ of ‘doing what you need to do’ was considered by some to be essential to them being able to leave secure care. Others described having moved to lower levels of secure care only to repeat therapies they had completed previously. For some this repetition of treatments could undermine the personal sense of progress that their otherwise progressive move had implied.

Self-perceived readiness to move on may or may not have been congruent with the views of clinicians; indeed, some who felt ready to move on perceived themselves as held back—notably by the clinician responsible for their care. Some described how their long stay in secure care had left them dependent on the system. Some felt they would never leave the secure care system, a view that was often explained in terms of repeated treatments and prolonged negative experiences, and these participants sometimes appeared indifferent to the prospect of moving. A lack of readiness to move on was also described by participants who felt comfortable and safe in their current unit, or who expressed concern that they were still a risk to others, or who worried that they could become unwell if they were to move.

In order to move on, many participants stressed the importance of keeping well, engaging with therapies and being able to go at their own pace. Some felt that progression could be hindered by staff being overly cautious due to negative perceptions held about them. For example, one participant described how the term ‘anxious’ is used by staff as a way of restricting patients:

*“… maybe people are anxious… it seems like a favourite word they’ve got, not just in this service, but in every service I’ve seen, as well….You get a lot more time added on …”* (NHS medium secure site)

At a more practical level, two participants—one deaf, and one who was in a wheelchair—were concerned that their potential for progression might be hindered by a lack of facilities suitable to their specific needs.

### Long stay stances

In the second phase of the analysis, the emergent themes described above were used to illustrate patterns of behavior that distinguished different participant narratives. This allowed four long stay stances to be defined which could collectively describe the positionality of our sample of participants. These stances, summarised in Table 1, were interpreted as *dynamic acceptance*, *static acceptance*, *dynamic resistance*, and *static resistance*.

**Table 1:** Summary of long stay stances

Theme	Long stay stance			
	Dynamic acceptance (14 participants)	Static acceptance (12 participants)	Dynamic resistance (nine participants)	Static resistance (five participants)
Outlook	Positive outlook towards being in secure care; believed their mental health had improved whilst in secure care	Positive outlook towards being in secure care; believed their mental health had improved whilst in secure care	Negative outlook towards being in secure care; feeling bored, restricted and frustrated	Negative outlook towards being in secure care; feeling bored, suffocated and a sense of pointlessness
Approach	Proactive approach; stressed the importance of keeping busy and making the most of their time by engaging in occupational activities and therapies	Proactive approach to occupational activities; less willing to take part in therapies that they found ineffective	Proactive approach to engaging in occupational activities and therapies that, although thought repetitive and pointless, would ultimately help them to move on	Passive approach to daily life; choosing not to engage in any occupational activities or therapies
Attribution (for their long stay)	Being unwell; their own behaviour	Their own behaviour; being on the wrong medication; being in a non-therapeutic environment	Risk-averse factors that left them feeling unable to prove themselves to staff	Interpersonal and structural factors outside their control
Readiness for change	Believed that they did not need to be in secure care; felt ready to move on to lower secure units	Believed that they were not ready to move on from their current unit	Believed that they did not need to be in their current unit but were stuck	Believed that they did not need to be in secure care but that they had no choice and so chose to remain

#### Dynamic acceptance

*“I’m glad I came here; it’s helped me out.” (NHS High secure setting)*

The fourteen participants who exhibited dynamic acceptance usually attributed their length of stay to personal factors or actions, such as disruptive behaviour or poor engagement. This group included participants who had moved from high secure to medium secure units, or to more independent wards within the same unit, and who believed that this progression was because of improvements to their mental health and, in turn, their behaviour. Three participants described how the worsening of their symptoms had led them to be transferred to higher levels of secure care. However, all believed these moves had helped them to get better.

Participants who displayed dynamic acceptance had an overall positive outlook on their current situation. All adopted a proactive approach, keeping busy and making the most of their time in order to maintain the progress they had made and to continue proving themselves to staff. Most emphasised how it was important to talk openly with staff on a regular basis, and they believed this had helped them to progress. Some participants had been granted community leave which they felt made their days more enjoyable. Many reported finding psychological therapies effective and engaged in recreational activities. Most felt they got on with other patients, but also noted that differences between themselves and others could prove challenging in such close proximities. Some described how they were able to better manage conflict with other patients having learnt to control their emotions, and as such tried to advise others to adopt a similar approach.

Participants who displayed dynamic acceptance often did not feel they needed to be at their current unit, explaining how they were ‘better’ or that there was no further treatment to undertake. They felt ready to move to a lower level of security, and often expected this to happen soon; some even anticipated rehabilitation back into the community. Nervousness was often expressed, however, that their chances of moving on could be jeopardised if they became unwell, or if they broke any rules. Most were also aware that the transition to another unit might be difficult as they would need to take on more responsibility for their own care whilst losing the support of familiar staff.

#### Static acceptance

*“It’s an as*-*you*-*were situation you know, continue to stay here and have treatment here. After all where would I go if I wasn’t here? See what I mean?”* (NHS medium secure setting)

Unlike other long stay stances, the twelve participants exhibiting static acceptance felt settled or safe in their current units and were not actively seeking to move. Although a majority felt that they did not need to be at their current unit, their acceptance was based on feeling safe or comfortable, or both. They took the view that if they continued with what they were doing at their own pace with regards to their treatment and good behaviour then ‘maybe’ they could move on. However, a minority who felt they continued to pose a risk reported that their current security level offered safety and containment, which some felt could probably not be bettered elsewhere.

This acceptance appeared to have a positive effect on their outlook towards their current situation. Most adopted a proactive approach in relation to their daily routine and participation in occupational activities; the importance of keeping busy through routine and structure was particularly emphasised. However, these participants tended to take a more passive approach to psychological therapies, and participated in these with some reluctance. They described feeling ‘fed up’ with therapy which they saw as ineffective. They attributed their length of stay to receiving the wrong diagnosis, inappropriate care or medication, or being in a non-therapeutic environment. Nevertheless, positivity towards that environment was maintained because most felt that their current ward provided for good relationships with other patients (who they often perceived as similar to them), and staff (who they felt understood and cared for them despite their reservations about therapy).

#### Dynamic resistance

*“So here we have a situation where now I’ve got it all complete and still stuck.” (Independent medium secure setting)*

Nine participants adopted a stance we describe as dynamic resistant. They attributed their long stay to risk-adversity within secure care units where they felt stigmatised and unable to ‘shake off’ (a) their offending history, and (b) incidents that had occurred whilst in secure care which were seen as being used to prevent them making progress long after the event. This perceived risk-adversity also impacted on their daily lives, with many describing how they felt staff were overly restrictive in response to their offending history. For example, one participant described being closely monitored when reading a newspaper, feeling that staff may judge and effectively pathologise his reading of certain content.

These participants had an overall negative outlook on their current situation and most felt restricted, frustrated and bored. Some acknowledged that at times this lead them to act out or become violent. Some even felt that their mental health had deteriorated. One participant reported self-harming as a way to avoid harming others. It was common for these participants to consider other patients to be ‘madder’ or ‘worse behaved’ than they were, and to report relatively little socialising compared with others. Most of the participants who exhibited dynamic resistance had received a diagnosis of personality disorder.

Despite their negative outlook, and a strong sense that taking part in both therapies and occupational activities was pointless and repetitive, most of these participants nevertheless adopted a proactive approach and reported engaging in therapies and other activities in the hope that it would ultimately help them to move on. They generally felt ready to leave their current units having completed therapies and ‘done what they were told to do’, but felt stuck in secure care—usually because of the risk-adversity of staff. In order for them to move on, this group felt that staff needed to trust them and, in turn, allow them to take on more responsibility, such as gaining more leave to demonstrate they could “*exist within the community without causing any harm to any other person.”* (Independent medium secure site)

#### Static resistance

*“I’m not gonna get out so I might as well stay here.” (NHS high secure setting)*

Five participants adopted a static resistant stance. They believed that they did not need to be in secure care, that the severity of their index offence had been exaggerated (one completely denied committing their index offence) and as such treatment was unnecessary.

These participants all shared a common belief that the secure care system worked against them, and they attributed the length of their stay to interpersonal and structural factors that were outside their control. For example, one participant felt that the reputation she had built up whilst being in secure care had restricted her from leaving their current unit. Another participant described having to undertake more treatment and therapies due to a moving of the hospital’s ‘goal posts’.

Of all those interviewed, participants who exhibited static resistance had the most negative outlook towards their current situation, describing feelings of boredom, suffocation and pointlessness. They adopted a passive approach to daily life, and almost all expressed disinterest and lack of engagement in activities or therapies. They chose to keep to themselves and did not socialise with other patients. Their relationships with staff were also described as poor. Some felt staff put unnecessary restrictions in place and two participants described feeling targeted by certain staff who they felt took pleasure in belittling them, for example, by dictating when they could eat or make a phone call. These participants did not feel they had any power to influence their propensity to move on from secure care, and described previous experiences when they felt ready and able to move on, only to be unreasonably denied. As a result, they believed their chances of ever leaving secure care were either remote or impossible; they had become resolved to stay, albeit with minimal engagement.

## Discussion

This study has provided important insights into the perspectives of patients who spend an extended period in secure forensic psychiatric care. Four key themes emerged illustrating the extent to which participants: (1) attributed the reasons for their long stay to personal, interpersonal or structural factors; (2) held a positive or negative outlook towards their current situation; (3) adopted a proactive or passive approach to daily life; (4) felt ready for a progressive move from their current secure care setting. Further analysis illustrated how each participant positioned themselves in relation to these themes determined the patients’ overall stance in relation to long stay.

### Acceptance, resistance and perceived locus of control

It is clear that some patients attributed their length of stay to factors over which they had neither control nor responsibility, whereas others acknowledged their own part in the process. This difference may be viewed in terms of acceptance, resistance and, importantly, their locus of control. The concept of health locus of control [42] can be useful in describing the extent to which individuals attribute their health and wellbeing to their personal actions (internalised), or to environmental circumstances and external agents (externalised). Similarly, in terms of moving on, those patients who internalised their reasons for long stay (acceptance) believed in turn that their abilities to move on were determined by their own behaviour, whereas those who externalised their reasons for long stay (resistance) tended to believe that their abilities to move on from secure care were determined by factors largely out of their control. Members of this latter, externalising, group tended to display considerable negativity which may in turn have impacted on their ability to move on, and a more external locus of control has been shown to be related to fewer periods of recovery in patients with psychosis [16].

It is well-established that psychiatric institutions also act as a form of control by defining normalcy and appropriate responses to diagnosed mental illness, such as the need for patients to demonstrate ‘insight’ and comply with treatment (e.g. [35]). This was reflected in both of the ‘acceptance’ long stay stances in this study. In contrast, the ‘resistant’ long stay stances described here are in keeping with observations that some individuals use their individual agency to reject psychiatric care and the role of a psychiatric patient [6]. These resonances with previous research suggest a degree of face validity to the findings from the current study.

### Continuity and consistency when moving through secure care

Although consistency in the care they received were important for many of the long stay participants interviewed, a significant number expressed their frustrations with having to repeat therapies once they had moved to other units, even if they had moved due to positive progress. This opinion was not dependent on the type of stance exhibited. This may explain why some participants found therapies pointless and ineffective, and why those with static acceptance and static resistance stances chose not to engage. Experiencing therapies repetitively has potential to negatively influence patients’ motivations to progress and limit their ability to move on from secure care. In the UK prisons system, a system of accredited therapies (such as the Sex Offender Treatment Programme) is in place so that if a prisoner has completed an accredited programme (which will be very similar across the prison service) there would be no need to repeat such programme in another prison. A similar system of accreditation of psychological treatments in forensic-psychiatric care could be considered.

Staff turnover was also perceived as causing problems for patients’ progress, and making efforts to minimise changes in key professional relationships are likely to be of particular importance in providing a stable and therapeutic environment.

Most participants displaying *dynamic resistance* had been diagnosed with personality disorder. Issues of continuity and consistency may be particularly important for such patients when moving through secure care, particularly because of their difficulties establishing trust toward others and sensitivity to perceived rejection [28]. This situation is not helped by the tendency for patients with personality disorder to be seen more negatively than other patients within a secure care setting [4] as well as in the community at large [5]. The lack of a suitable step-down facility may also delay discharge from a secure forensic unit, and medium secure units may be reluctant to accept patients with personality disorder because they lack the infrastructure to treat this patient group [37].

Some long-term patients may not wish, or indeed be able, to move on. It may be more appropriate for this subgroup to concentrate on attempting to improve their quality of life at the moment where they are. A more settled, specific long stay environment could provide more opportunities for patients to take part in fulfilling activities (such as cooking or day trips) without being restricted by others who are less well or more disruptive than themselves.

### Motivations to engage and readiness for change

Whether participants were actively trying to progress (dynamic stance) or not progress (static stance) can be interpreted using the concept of ‘modes of adaption’ [43]. This differentiates motivation to change (the end result) and motivation to change in a particular way (the means of that change).

Participants who displayed *dynamic acceptance* were motivated to engage in therapies with the hope of bettering themselves with a view to eventually moving on—a process that has been termed optimistic compliance. In contrast, those who displayed *dynamic resistance* and who chose to engage in therapies as a means to an end reflects an instrumental mode of adaption, and it was apparent in the interviews that ‘doing what you need to do’ was the mechanism through which some participants sought to leave secure care.

This is relevant here as it has been suggested that a key issue lies not in the changes that offenders wish to seek (e.g. leaving secure care) but in the way in which they seek those changes [23]. Overly focusing on leaving secure care may hinder patients’ progression when they do not address the reasons why they are in forensic care [17].

Jones [21] argued that individuals tend to move between different adaptive modes as they go through the treatment process. This resonates with those who displayed *static resistance* who had built up their hopes too often in the past only to be ‘knocked back’ due to perceived rule changes. This demonstrates how an initial instrumental approach could fall into a state of intransigence where participants come to reject therapeutic interventions through cynicism or indifference. Similarly, those who displayed a stance of *static acceptance* were also not actively trying to leave secure care. They, however, tended to adopt a ‘ritualistic’ mode of adaption when engaging in occupational activities where there was little investment towards an end goal. Their desire to stay in their current unit may be associated with previous negative experiences where they had either been kept in a unit due to not receiving the right care or had been moved to other units for which they did not feel ready.

It is therefore important to consider how to achieve readiness for change in long stay patients who lack motivation to progress. Aspects for consideration include the patient’s judgement of the need for change, the possibility of changes occurring, the patient’s belief in their ability to change, and stating an intention to change within a particular time frame [25]. A focus on what will help to motivate the patient from their own personal perspectives may also help to reduce tokenistic engagement.

### Perceptions of risky behaviour

It has been suggested that the need by professionals to maintain safety can often result in a culture of control, which in turn leads to risk-averse, defensive practice and, ultimately, over control [22]. For patients exhibiting resistant stances, the restrictions put in place to prevent risky or disruptive behaviour were seen by them as unnecessary control and ultimately as the source of what caused them to become frustrated and act out. Differences in perceptions of risky behaviour resulted in dissonance not only between participants and staff but also with other patients. It was common for these participants to describe not being able to get on with other patients whom they perceived to be ‘madder’ or more disruptive. The extent to which behaviour was perceived to be risky may have a negative impact not only on patients’ relationships with staff and other patients, but also on the way in which patients decided to manage this behaviour.

### Limitations

We identified three limitations to the study. First, all participants had been in secure care for prolonged periods, and this sometimes made it difficult for them to recall the details of events that occurred many years previously. Second, the study’s epistemological position dictated that it was neither appropriate, nor was it our intention, to make any judgement on any participant’s level of insight. However, it was apparent that the extent to which participants were aware of being unwell or of their index offence varied, and this may have affected their perceptions and experiences of secure care. Third, although the 40 participants were purposively sampled from a long stay population identified by a large epidemiological study of secure care, we cannot exclude the possibility that differing views and perspectives may have been held by patients who refused to participate, or who were accommodated at sites not sampled, or for whom the clinician in charge of their care did not give permission to take part.

## Conclusions

Patients who spend an extended period in secure forensic psychiatric care form a heterogeneous group in terms of how they attribute the reasons for their long stay, how they view their future, and their motivation to progress. Given this diversity and the complexity of the issues which have resulted in their long stay and their difficulties in moving on, careful long-term planning for this patient group is warranted. Such planning should take account of:

- The differing stances adopted by long-term patients in secure care, interpreted in this study as dynamic/static acceptance and resistance.
- The need for clinicians and other health professionals to work together between units to prepare patients for transfers.
- Minimising changes in key professional relationships which are of particular importance in providing a stable and therapeutic environment for this patient group.
- The need for collaboration between units to avoid patients repeating treatments.
- Considering how to achieve readiness for change in long stay patients who lack motivation to progress and tend towards tokenistic engagement.
- Accepting that some long-term patients may not wish, or indeed be able, to move on, and that refocusing on quality of life may be more appropriate.

## Supplementary information

**Additional file 1.** The questions in the interview guide were designed to explore reasons for the patients’ long stay, their current situation and their feelings about progression through the system.

## Data Availability

Data is not available since it could compromise the individual privacy of participants.

## Abbreviations

NHS: National Health Service
MHA: Mental Health Act
