# Supplementary material for: The experience of long stay in high and medium secure psychiatric hospitals in England: qualitative study of the patient perspective
Source: Int J Ment Health Syst. 2020 Mar 30;14:25. doi: 10.1186/s13033-020-00358-7 (PMC7104497; doi:10.1186/s13033-020-00358-7)
Supplement: Supplementary file 1 — Additional file 1. The questions in the interview guide were designed to explore reasons for the patients’ long stay, their current situation and their feelings about progression through the system. [file 13033_2020_358_MOESM1_ESM.docx]

**Interview agenda and topic guide**

***Introductions***

- **Thank you** for agreeing to help in this study. By participating in this study, you will help to improve our understanding of staying in secure-care. I’d like you to help us to understand what it’s like being in secure care. If there are any questions you don’t want to answer that’s fine and if you want to stop or take a break that’s entirely up to you. Do you have any questions before we start?
- **Why you have been invited –** We are interested in your experience of staying within the unit because you have been in secure care for a while - this gives you good insight into what it’s like to stay in a secure hospital.
- **What we will cover –** Talk through the interview topics – make clear, but be brief.
- **Icebreakers- let them lead!** E.g. would you prefer to start by talking about your current situation or to go back a bit and describe how you first came into this hospital?

***Main Interview-***

- **What it’s like** to have stayed in secure-care a long time
- **Why you think you have stayed** in secure-care for this long
- **What you expected** ‘vs’ how you feel now
- Whether you think you **need to be at the current level of security**
- What it’s like **moving between** different levels of secure-care
- How you think **life could be improved** for you
- What you think about the potential for **specific long-stay units**

***Ending the interview***

- **Thank you** – Thank you for your time, it’s been really valuable and interesting.
- **End questions** – Is there anything else you’d like to add or that you think we’ve missed? Do you have any other questions about the study?

**Icebreaker questions:**

1. Could you describe to me what a typical day looks like? / Could you briefly describe your day-to-day experiences of being in secure care?/ Can you tell me a little bit about what it’s like to be in here?
2. Let me take you back a bit… Could you describe how you first got here? (if transferred from other secure-unit)/ Can you tell me what life was like before you came here?

**Part 1: Experiences of Long-stay**

**1)** How does it feel to have stayed in secure care for a long time?

-If someone asked you to describe what it’s like to be in secure care for a long time, what would you say to them? What helps to pass the time?

-Can you think of anything that has helped you during your stay which you feel you don’t need now?

-Can you think of anything that has helped you during your stay which has now stopped even though you wish it hadn’t?

**2)** Why do you think people stay so long in secure-care? And does this apply to you?

- Did you know how long you would be staying in a secure hospital for? / Has anyone ever spoken to you that you would likely be in a unit for a long time – potentially life-long?

**3)** What did you expect when you first came into the unit? Have your expectations changed? / If so, how do you think this has impacted upon your stay?

-Since you’ve been here, do you think anything has happened while you’ve been here that you think has meant, ‘I’ve stayed longer because that happened’? For example, how did your last tribunal go?

**4)** –Can you describe the level of security here?

Do you feel you need to be at this current level of security?

-If yes, why is this?

- If no, where do you think you should be and why?

-What do you think could be improved by moving to a different level of secure-care?

**5)** Could you describe your experiences (if applicable) of moving between different levels of secure care?

- How did it happen?

- Did you feel prepared?

- Why do you think you moved?

-Could you describe any similarities/differences in these settings?

**6)** **Pathways** - Has anyone talked to you about care pathways?

- Do you know whether you are in one? Has it been explained to you? If so, what do you understand by this?

- Has being in one made any difference/changed your experiences of care?

- - Strengths/positives of current services
  - Weaknesses/negatives of current services
  - Are there any gaps in the services that are currently provided to them?

**1A: Staff/patient interactions**

- How do you get on with staff and other patients? (More general)

-How were members of staff involved?

- Did you find their involvement helpful?

**1B: Involvement in decision making**

- Were you informed of this? If so, did you understand what you being told?

- Were you involved in the decisions made or, did you feel decisions were made for you?

- Do you feel informed about what is available to you in terms of your care/stay in the unit?

**1C: Impact on Quality of life (QoL)**

- How did this impact upon your QoL/ your needs/ your recovery?

- Or, (more generally) how has being here for a long time impacted upon your QoL?

- How could this aspect of your life be improved/ worsened?

-QoL prompts (if participants do not mention any specific examples):

- Building and sustaining relationships
  - - - Family
      - Friends
      - Intimate
      - Other patients
      - Staff
    - Autonomy, Level of restrictiveness
    - Finances (and financial decisions)
    - Occupation / meaningful activity (including interests, studies, hobbies, work, self-care)
    - Education and work skills
    - Physical wellbeing
    - Dignity, respect, privacy
    - Having meaning in what you do / Sense of meaning in life
    - Religion/spirituality

Do you think that your perceived quality of life could be improved by being moved to a different level of secure care/ setting?

**1D: Impact on Mental Health**

-How do you think this impacted upon your mental health?

- Did you feel that your mental health improved/ got worse?
- Why do you think this improved your mental health/ made you feel better?
- Why do you think this made you unwell/ caused you to relapse?

**Part 2: Future**

**How life could be improved**

- - Where do you think you will be in 2 / 5 years’ time?
  - When is your next tribunal? What is your expectation of it?
  - What does moving on mean/look like to you? / Do you have any expectations for progression and/or change?
  - What do you think helps/stops you from moving on (Organisational/structural/procedural changes?)/ what do you think would help you to get to where you want to be?
    - How/when did you feel that you were being helped/ stopped from moving on and who by?
- Having been here for a long time, how do you feel about being here with other patients who have not been here for as long as you?
- Have you seen others move on from the unit and how did that make you feel?
  - Do you have any concerns about your future? Does being in secure care contribute to this? Is so, in what way?
  - Is there anything you would like to stay exactly how it is now? / Is there anything you would not change about your current situation/ circumstances?
  - What could be done to improve your current situation/circumstances? / Is there anything that could help you feel more hopeful for the future?
- Do you think that you will leave the unit (ever)?
- Do you think that you will live in the community again (ever)?
